# Ethical issues in using the internet to engage participants in family and child research: A scoping review

**DOI:** 10.1371/journal.pone.0204572

**Published:** 2018-09-27

**Authors:** Stacey Hokke, Naomi J. Hackworth, Nina Quin, Shannon K. Bennetts, Hnin Yee Win, Jan M. Nicholson, Lawrie Zion, Jayne Lucke, Patrick Keyzer, Sharinne B. Crawford

**Affiliations:** 1 Judith Lumley Centre, La Trobe University, Melbourne, Victoria, Australia; 2 Murdoch Children’s Research Institute, Melbourne, Victoria, Australia; 3 Parenting Research Centre, Melbourne, Victoria, Australia; 4 Queensland University of Technology, Brisbane, Queensland, Australia; 5 Department of Communications and Media, La Trobe University, Melbourne, Victoria, Australia; 6 Australian Research Centre in Sex, Health and Society, La Trobe University, Melbourne, Victoria, Australia; 7 School of Public Health, The University of Queensland, Brisbane, Queensland, Australia; 8 La Trobe Law School, La Trobe University, Melbourne, Victoria, Australia; University of Illinois at Urbana-Champaign, UNITED STATES

## Abstract

**Background:**

The internet is an increasingly popular tool in family and child research that is argued to pose new ethical challenges, yet few studies have systematically assessed the ethical issues of engaging parents and children in research online. This scoping review aims to identify and integrate evidence on the ethical issues reported when recruiting, retaining and tracing families and children in research online, and to identify ethical guidelines for internet research.

**Methods:**

Academic literature was searched using electronic academic databases (Scopus, PsycINFO, Embase, ERIC, CINAHL and Informit) and handsearching reference lists for articles published in English between January 2006 and February 2016. Grey literature was searched using Google to identify relevant ethical guidelines.

**Results:**

Sixty-five academic articles were included after screening 3,537 titles and abstracts and 205 full-text articles. Most articles reported using the internet to recruit participants (88%) with few reporting online retention (12%) or tracing (10%). Forty percent commented on ethical issues; the majority did not discuss ethics beyond general consent or approval procedures. Some ethical concerns were specific to engaging minors online, including parental consent, age verification and children’s vulnerability. Other concerns applied when engaging any research participant online, including privacy and confidentiality, informed consent and disparities in internet access. Five professional guidelines and 10 university guidelines on internet research ethics were identified. Few academic articles (5%) reported using these guidelines.

**Conclusions:**

Engaging families and children in research online introduces unique challenges requiring careful consideration. While researchers regarded themselves as responsible for ensuring research is conducted ethically, lack of use of available guidelines and limited academic literature suggests internet research is occurring without suitable guidance. We recommend broad dissemination of ethical guidelines and encourage researchers to report the methodological and ethical issues of using the internet to engage families and children in research.

## Introduction

Parents and children in contemporary society are facing new and multiple demands that may affect their wellbeing, and limit parents’ capacity to support their child’s healthy development [1]. High quality research is key to understanding these changes and to providing a sound evidence base for the development of policies and programs to support families, parents and children. Being able to engage families from diverse backgrounds and in sufficient numbers is critical to the completion, scientific validity and financial viability of such research [2]. However, a major research challenge is the ability to attract and retain participants over time to assess long-term outcomes [3], particularly within the context of increasing family time pressure and population mobility.

Engaging participants in research can involve: *recruitment* (strategies to invite potential participants and enrol them into research); *retention* (strategies to ensure ongoing participant involvement to reduce attrition); and *tracing* (strategies to find and re-connect with participants who have been lost to follow-up). Conventional approaches to engage families and children in research (e.g., telephone calls, mail-outs, print media, face-to-face) are not only expensive and labour intensive [4, 5], but are increasingly ineffective in the face of contemporary mobile populations and changing communication patterns [6]. For instance, the use of random digit dialling to recruit participants is less effective as many families now live in households without a fixed telephone [7]. Recruiting families via address-based sampling is also difficult in the context of increasing residential mobility, which is high among young and socially disadvantaged families (e.g., experiencing poverty, unemployment, relationship breakdown) [8, 9] who are often the target of research. Maintaining contact with mobile families can be challenging in longitudinal research, especially if there are lengthy periods between data collection waves or if a prospective follow-up study was not part of the initial research design [10].

Increasingly, child and family researchers are using online methods to recruit, retain and trace participants [6, 11–13]. In developed countries, internet access is almost universal among households with children, with most connecting online via mobile or smart phones [14, 15]. Children and adolescents have among the highest rates of internet use and frequency of use compared to older age groups, and social media use is ubiquitous among adolescents [16]. Parents are also increasingly online [17]. More parents access Facebook on a daily basis than non-parents, with usage tending to increase during the transition to parenthood [18, 19].

As a research tool, the internet provides alternative strategies to reach participants where they ‘live’, and presents an ostensibly efficient and cost-effective solution to the challenges associated with conventional methods of participant engagement [20]. For example, study invitations can be advertised on websites or circulated via email to a large audience quickly and at little expense. Advertisements on social networking sites such as Facebook can be specifically targeted to identify eligible participants based on their personal characteristics or interests. Evidence suggests that child and parent samples can be recruited more efficiently and at a lower cost online than offline, and may result in a sample that is representative of the target population, or which has similar representativeness to studies using offline recruitment methods [21–23]. The internet also has potential to reduce attrition and non-response bias in longitudinal research with children and parents [13, 24, 25]. Posting on study websites or directly messaging participants on social media can maintain contact with study cohorts and send calls-to-action at appropriate times (e.g., for measure completion), while internet search engines and the search features of social networking sites can be used to trace participants who have been lost to follow-up.

Given the ubiquity of the internet and smartphone technology, the public availability of online communities and the potential cost and time efficiencies, it is unsurprising that family and child researchers are increasingly harnessing the internet to engage participants. As with all new research methodologies however, consideration needs to be given to the ethical issues surrounding its use. It has been argued that maintaining privacy and anonymity and obtaining informed consent are more complex in online research than offline research [26]. As a relatively new research tool, engaging participants online may also present issues that may be novel or less apparent. For example, the internet, and particularly social media, introduce new ways to access data and personal information. This raises questions regarding the ethical appropriateness of accessing digital data or online profiles for research purposes, and how the ethical principles embodied in codes of conduct (e.g., [27]) are upheld.

Ethical issues may be particularly pertinent in online research with parents and children. Such research may be sensitive in nature and involve vulnerable populations. Communicating with and engaging minors in an online setting can present further challenges, such as how researchers accurately determine a child’s capacity to consent to participate and the process of obtaining parental consent. Within this context, it is imperative that researchers, ethics committees and institutional review boards are aware of the advantages, limitations and ethical challenges of recruiting, retaining or tracing participants online. While discourse on internet and social media research ethics in family and child research is emerging [4, 28], this may not be empirically based. In addition, there is a lack of definitive national regulatory guidance on internet research ethics. To date, little research has systematically assessed the ethical issues encountered or considered by those using the internet to engage participants in child and family research. It is unclear what ethical issues are considered by researchers in this setting and whether these ethical concerns differ from those in offline research, or in research not involving children and parents. It is also unclear what ethical guidance family and child researchers follow when recruiting, retaining or tracing participants online.

To address these gaps, a scoping review was conducted. Scoping reviews aim to synthesise and narratively integrate evidence when there is a large and diverse body of literature that has not been comprehensively reviewed [29]. They aim to map the extent, range and nature of research, and identify key concepts and gaps in the literature to inform future work [29, 30]. Scoping reviews differ from systematic reviews, in that they are often broader in focus, include a range of study designs and methodologies, and typically do not appraise study quality. They differ from narrative or literature reviews as they follow rigorous, systematic methods to identify literature and require analytical re-interpretation of evidence [30].

This review was conducted to gain an understanding of the ethical issues associated with the use of the internet to engage parents, children and families in research, and to identify available resources to support the ethical conduct of family and child researchers in the context of online research. The review aimed to assemble and summarise the ethical concerns and considerations reported in available literature and identify ethical issues unique to using the internet to recruit, retain and trace families and children in research. A systematic search of academic literature was conducted to answer the following research questions: (1) How is the internet used to recruit, retain and trace participants in child and family research?; (2) How are ethical and consent procedures reported?; and (3) What ethical concerns are identified? In addition, a general grey literature search was conducted to determine: (4) What ethical guidelines are available and being used to inform internet research?

## Methods

A multi-disciplinary research team was established to undertake the review, with expertise in child and family research, public health, psychology, media and communications, and law. The scoping review was conducted according to a 5-stage framework [29–31]: (1) identifying the research questions; (2) identifying the search strategy; (3) study selection; (4) charting the data; and (5) collating, summarising and reporting the results. These steps were applied separately to searches of the academic and grey literatures.

### Academic literature search

To address Research Questions 1 to 3 above, academic literature was searched using four categories of keywords: *engagement strategy*, *internet*, *ethics* and *focus sample* (Table 1). Boolean search operators “AND” and “OR” were used to combine keywords between and within categories. Searches were conducted in the following electronic databases selected to ensure maximum coverage of family, child and internet research: Scopus, PsycINFO, Embase (includes Medline), ERIC, CINAHL and Informit. As the Scopus search generated over 70,000 initial hits, results were refined by applying additional search limits (see S1 File).

**Table 1:** Keywords for the literature search.

Engagement strategy	Internet	Ethics	Focus sample
recruit*	internet*	ethic*	famil*
retention	technolog*	privacy	parent*
retain*	social network*	confidential*	child*
engag*	social media	informed consent	teen*
track*	web*	institutional review board*	adolescen*
trace*	computer*		
tracing	Facebook		
follow-up	online		
attrition			

Articles were eligible for inclusion if they were written in English, published between January 2006 and February 2016, and used the internet (e.g., social network sites, websites, email, listserv, blogs, forums, applications, online research participant registries) as a method for recruiting, retaining or tracing families, parents and/or children in research. Studies that used the internet for data collection but not participant engagement were excluded. Studies that used online residential listings were also excluded as these listings are typically used for compiling subsequent mail-out or door-knocking recruitment strategies. Included articles could be original research, reviews, discussion papers, outcomes papers, protocol papers or editorial letters. Books, book chapters and thesis dissertations were excluded. Published conference abstracts were used to search for subsequently published full-text articles, which were included if they met the inclusion criteria.

Upon completion of the search, duplicate articles were removed and search results were screened by title and abstract for eligibility. Articles passing the abstract screen were then retrieved in full-text and further screened for eligibility. Screening of abstracts and full-text articles was undertaken by a primary reviewer (NQ) and confirmed by a second reviewer (SH). Conflicts were resolved by consensus with a third and fourth researcher (NJH, SBC).

Articles were summarised, and characteristics were charted, including: author(s), year of publication, geographic location, article type, study participants, method(s) of participant engagement, type of internet technology used, study approval and consent procedures, ethical considerations and concerns, and authors’ use of ethical guidelines and resources. Methods of participant engagement were defined as follows: *recruitment*—strategies to initiate contact and invite potential participants; *retention—*strategies to maintain contact with participants in longitudinal research; and *tracing*—strategies to find and re-establish contact with participants in longitudinal research who have been lost to follow-up. Reference lists of full-text articles were searched and eligible articles charted. Each article was analysed by a primary reviewer (SH) and extracted data confirmed by a second and third reviewer (NJH, SBC). A narrative account of key findings is presented according to each ethical consideration identified.

### Grey literature search

Ethical guidelines and recommendations produced by governments, research institutes or professional associations are documents classified as ‘grey literature’. This term refers to reports, theses, factsheets, websites, policy documents and other information produced by government, academics, business and industry in print and electronic formats. These documents are not controlled by commercial publishing [32], may not be peer-reviewed and most are not included in academic databases. Grey literature published online can be located through internet searches [33].

To address Research Question 4, an internet search was conducted to identify guidelines to support researchers’ and human ethics committees’ ethical conduct of internet research. A general internet search was performed with the popular web search engine Google (www.google.com) using the following search terms: (ethic*) AND (recommendation* OR guide*) AND (internet OR "social media" OR online) AND (research). The first 100 hits (as sorted for relevance by Google) were screened by title and text and potentially relevant results were viewed. Guidelines were included if they were published from January 2006 to February 2016 and focused on any internet research methods. Information about each resource was charted, including author(s), affiliated institution(s), year of publication, format and purpose, comprehensiveness regarding the research methods raised, and the ethical considerations and recommendations discussed, including ethical issues specific to family and child research.

## Results

### Summary of search process and results

**Fig 1:**
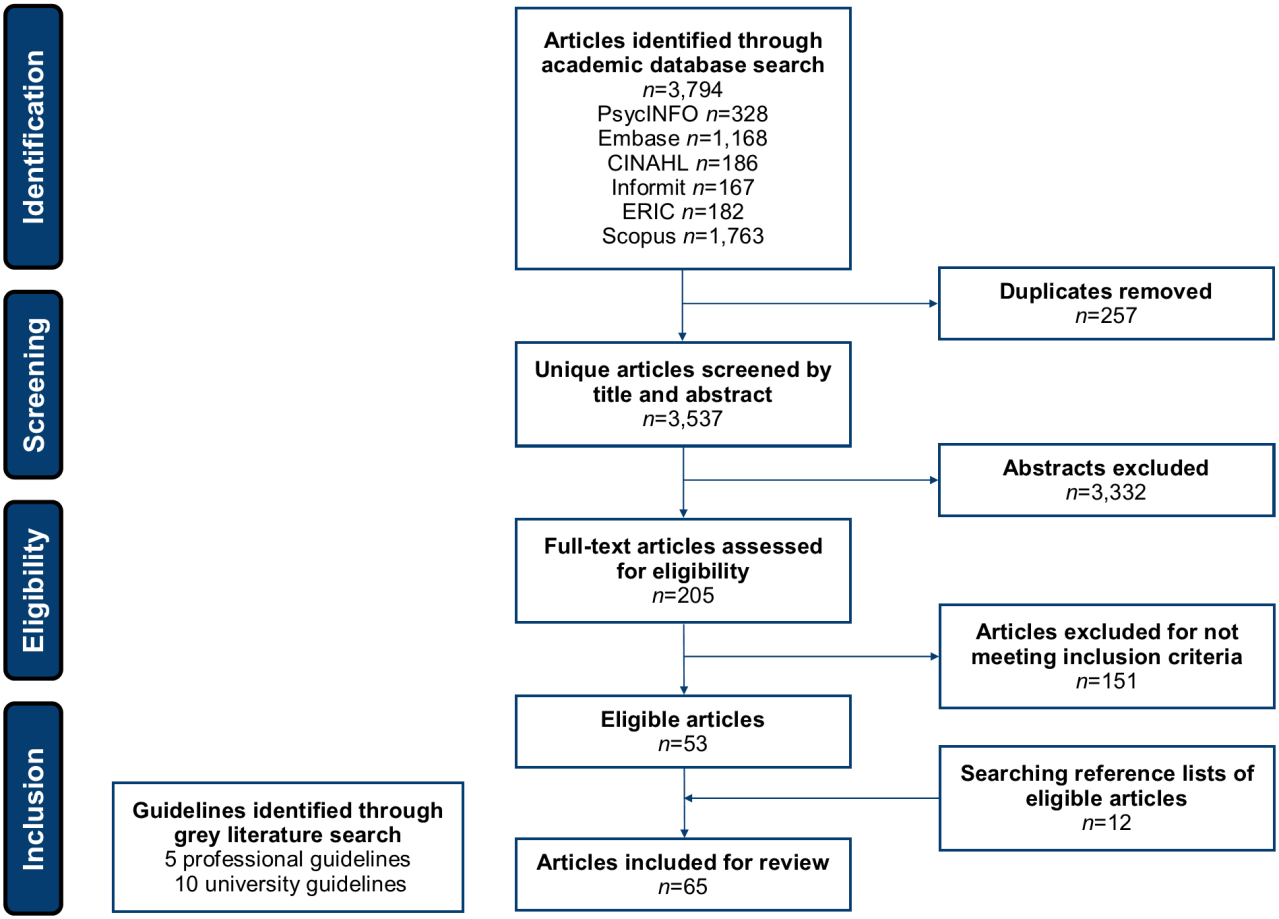
Search process and results.

The search process and results are summarised in Fig 1. Searching academic databases identified 3,794 abstracts, with 3,537 remaining following the removal of duplicates. Preliminary screening of abstracts identified 205 articles for full-text screening, of which 53 articles met the inclusion criteria. Hand searching reference lists identified 12 additional relevant articles, resulting in a total of 65 academic articles for inclusion in the scoping review (see S2 File). Five professional guidelines and 10 university guidelines specific to internet research ethics were identified by Google search.

### Academic literature article characteristics

The majority of identified academic articles were published after 2011 (*n* = 47, 72%) and originated from the USA (*n* = 24, 37%), UK (*n* = 12, 18%), Australia (*n* = 11, 17%) and the Netherlands (*n* = 9, 14%). Most were original research (*n* = 58, 89%), including four research protocols (6%) and 10 case studies (15%). The vast majority of original research papers reported using the internet to *recruit* research participants (*n* = 51/58 articles; 88%). Using the internet to *retain* participants was reported in seven articles (12%; six also used the internet for recruitment) and to *trace* participants in six articles (10%). Four discussion papers (6%), two systematic reviews (3%) and one letter to the Editor were identified, and these primarily focused on participant recruitment. Overall, one third of the articles addressed or described specific ethical issues of engaging participants online (*n* = 26/65, 40%). The remainder reported using the internet as a research tool but did not discuss ethics beyond reporting general consent or ethics approval procedures.

The participant samples for the original research articles were predominantly youths (*n* = 20/58, 34%; typically ranging in age from 15–25 years) or adolescents (*n* = 13/58, 22%; typically ranging in age from 11–18 years). Six studies used mixed samples of child/adolescent or adolescent/adult participants (*n* = 6/58, 10%) and four longitudinal studies (*n* = 4/58, 7%) traced children into adulthood. The participant samples for the remainder were parents (*n* = 11/58, 19%) or families (e.g., parents and children; *n* = 4/58, 7%).

### How is the internet used to recruit, retain and trace participants in child and family research?

Just over half of the research articles reported using a combination of offline and online methods to engage participants (*n* = 32/58; 55%), with 45% (*n* = 26/58) using online methods exclusively. As summarised in Table 2, websites, Facebook and email were the most commonly used internet technologies. The use of websites or email for participant engagement appeared to be stable across the study inclusion period (2006–2016). Studies using social networking sites, such as Facebook, to engage participants were first published in 2011 and were used consistently thereafter.

**Table 2:** Type and frequency of internet technology used to recruit, retain and trace research participants.

	Recruit (*n* = 51) *n (%)*	Retain (*n* = 7) *n (%)*	Trace (*n* = 6) *n (%)*
Combination of online and offline methods	26 (51)	4 (57)	5 (83)
Internet technology: multiple types	6 (12)		
Internet technology: one type only^a^	19 (37)	3 (43)	1 (17)
Website: specific (website named)	9 (18)		1 (17)
Website: general (type of website described)	12 (24)		
Website: no detail	6 (12)		
Study website	4 (8)	1 (14)	
Forum, blog or discussion board	9 (18)		
Facebook	18 (35)	1 (14)	5 (83)
Other social networking site (named)	5 (10)		5 (83)
Social networking site (unspecified)	5 (10)		
Email	14 (27)	5 (71)	
Search engine	1 (2)		1 (17)
Online recruitment service	2 (4)		

^a^ Studies engaging participants by advertising on multiple websites or on multiple forums are regarded as using one type of internet technology.

For participant *recruitment*, advertisements and posts on websites were the most commonly used online method (*n* = 27/51; 53%). The level of detail provided about the websites used varied considerably (see Table 2). Nine articles (18%) named the website, 12 (24%) gave a general description, six (12%) provided no information, and four (8%) created a study-specific website for recruitment. Facebook was the most common social networking site used for participant recruitment (*n* = 18/51; 35%). Five articles (10%) reported the use of another social networking site (e.g., Twitter, MySpace) and five (10%) did not name the social networking site used. Email was the next most common online method for recruitment (*n* = 14/51; 27%), with invitations sent to members of electronic mailing lists, listservs, research panels and registries. Several studies sought to recruit participants by posting study information on blogs, forums and discussion boards (*n* = 10/51, 20%). Less common recruitment methods included: respondent-driven sampling online, where existing participants were asked to invite their friends to participate (via email, Facebook or an unspecified method); dissemination of the study invitation through online social networks (snowballing); and the use of researchers’ own personal online networks.

Few studies reported on the success of using the internet to recruit participants in research compared to offline methods. Of the 25 studies that reported using both offline and online methods, less than a third (*n* = 7; 28%) commented on how their sample was sourced or the relative success of each recruitment strategy. Four studies found online methods yielded more enrolled participants than offline methods [34–37] and three studies reported the opposite [38–40]. Recruitment sources and success rates were reported by half of the studies using either a single (*n* = 8/18, 44%) or multiple methods of online recruitment (*n* = 3/6, 50%).

For the *retention* of participants in longitudinal research, five studies used email to keep in contact (*n* = 5/7, 71%), one used a study website and one became ‘friends’ with participants on Facebook. Four studies (*n* = 4/7, 57%) employed both offline (e.g., mail, phone, SMS) and online retention methods [34, 41–43]. Again, the success of online versus offline methods for retaining participants was poorly reported. Only one study provided details on retention rates using email reminders and a subsequent mail-out to non-responders [34] and concluded that multiple reminders using online and offline modes of contact improved overall response rates. Two studies reported retention rates of the cohort overall, rather than by the method used to maintain contact [41, 42] and one study did not report retention rates, yet remarked that Facebook was a more effective retention tool than email [43].

Using the internet to *trace* participants who had been lost to follow-up was reported in six studies. The majority (*n* = 5/6; 83%) combined this with offline methods (e.g., electoral roll, mail, phone, school roll). Most commonly, online tracing was done via social networking sites (e.g., Facebook, Myspace, Friends United) using the platform’s messaging feature to send private messages to potential participant matches, identified by the information publicly displayed on their profile. Researchers also traced participants using internet search engines or by posting messages on relevant websites requesting participants to contact them. Half of the six studies using internet tracing reported on the success of this compared to offline tracing methods [44–46]. All three concluded that social networking sites were a valuable tool to trace and reconnect with participants, with Masson et al. [44] reporting a higher response rate for participants traced via Facebook than those traced using the electoral roll and contacted via post. Conversely, a fourth study commented that social media tracing was less successful than offline methods but did not provide tracing success rates [47].

### How are ethical and consent procedures reported?

The majority of research articles reported obtaining study approval from an ethics committee or institutional review board (*n* = 46/58; 79%). Ethical approval was not specifically mentioned in 10 articles (17%). One article stated that the researchers did not seek ethical approval [48] and another was noted as exempt from ethical review [49].

Informed consent was obtained online in approximately one third of studies via electronic signature, email or online checkboxes (*n* = 19/58, 33%) and one study obtained consent via text message [50]. Despite the online nature of recruitment, offline methods of informed consent (verbal or post) were also common (*n* = 16/58, 28%). ‘Implied consent’ was reported in four studies (7%) [35, 48, 51, 52] where the researchers regarded that participants had provided consent by the act of participating in online data collection. Some articles did not mention whether (*n* = 12/58, 21%) or how (*n* = 5/58, 9%) consent was obtained.

Of the 40 studies involving adolescents or children, 12 (30%) stated that parental consent was obtained if participants were younger than 16 or 18 years with eight of these studies also obtaining child assent. Parental consent was waived in nine studies (23%) involving adolescents (aged 11–17 years). Reasons provided related to the anonymous nature of the study, the study’s minimal risk, the perception that parental permission would deter adolescent participation, to prevent participants disclosing sensitive information to their parents (i.e. sexual identity) and national age of consent laws [20, 48, 52–55]. Sensitivity of the topic alone (e.g., adolescent mental or sexual health) had little bearing on whether parental consent was sought. Nineteen articles (48%) involving adolescents or children did not discuss parental consent procedures.

Of the six studies that used the internet to trace participants, three had previously obtained consent for follow-up, without specifying the method of contact in this consent [46, 56, 57]. Two studies did not have formal permission to re-contact individuals, likely due to the length of time elapsed (10–50 years) since initial contact [44, 47], and one study did not report consent procedures [45].

### What ethical concerns are identified?

Sixty percent of the reviewed articles did not comment on any ethical concerns associated with using the internet to engage participants. The remaining articles (*n* = 26/65) discussed one or more ethical considerations. As summarised below, some of these issues were specific to family and child research online (e.g., parental consent, age verification, children’s vulnerability) while others are more broadly applicable (e.g., privacy and confidentiality, disparities in internet access).

#### Parental consent

Several articles noted that recruiting children into research online and obtaining parental consent can be ethically complex and logistically difficult [36, 58–61]. The required dialogue between researcher, child and parent is challenging to achieve in the absence of face-to-face communication [36, 54]. Researchers must adequately inform parents, gain their trust, and reassure them that the research is legitimate and reputable [60, 62]. This may be facilitated by personally contacting parents or by providing them access to multiple sources of study information (e.g., study websites and social networking profiles) [36]. One review article suggested utilising video or audio recordings to enable parents and children to provide consent/assent online [63]. Study authors noted that further information and guidance on best practices of gaining parental consent online are required [11, 54].

Obtaining parental consent via post or email for children engaged in research online had several limitations. One study reported that very few children returned signed consent forms by post [60], possibly due to the inconvenience and cost of this process. There is also limited capacity for researchers to ensure that participants are sufficiently informed [59], and a risk of parental consent forms being fraudulently signed by young participants or non-parents [34, 59, 64]. One study reported excluding young adolescents (13–16 years) due to concerns that they might fraudulently complete the online parental consent forms themselves [34]. Given these barriers, Henderson et al. [59] recommended seeking child and parent consent via telephone. This allows researchers to separately test child and parent speak understanding of the study procedure, risks and benefits, and enables verification of the child’s age with the parent. Amon et al. [11] alternatively proposed that researchers interested in recruiting young people via social media may benefit from directly targeting parents instead of children.

Several other methods were used to obtain consent for child participation. Boydell et al. [62] encouraged participants (aged 14–18 years) to discuss the study with their parents and then included a tick box in the online consent form for adolescent participants to declare this. One study performed a mature minor assessment of interested respondents aged under 18 years by telephone [20, 65, 66]. This involved the researcher assessing a participant’s age, schooling, general maturity, and ability to understand the study, to determine their capacity to provide informed consent.

In some studies involving children and adolescents, waiving parental consent was considered a methodological and ethical necessity. For example, Moinian [58] conducted an ethnographic study of children’s diary entries within an online community to explore the online activities that children engaged in outside of their parents’ knowledge or control—a topic that made “the process of obtaining parents’ consent practically impossible” [58, p. 56]. In other studies, parental consent was waived if parental permission was likely to deter participation [54], or would put participants at harm. For example, parental permission was waived in two US studies involving gay and bisexual adolescents as young as 13 years [52, 55] as it was considered that young people may be placed at risk by disclosing their sexual identity to their parents. Some studies noted that where parental consent was required by law, younger participants were under-represented due to their reluctance to seek parental permission [61, 67, 68] or due to parents not providing consent [69]. These articles debated the age at which an adolescent should be able to give informed consent without parental permission, although this was not specifically discussed in the context of online research.

#### Age verification

Verification of participant identity and age presents challenges in online research [53, 59, 62]. This was of particular concern in studies involving child and adolescent populations, with one article commenting that “attempts can, and arguably should, be made to verify the age of participants [in online research with young people]” [62, p. 13]. Verifying participant age was also pertinent to research with adults, as minors may participate without the knowledge of the investigators [64].

To verify participant age, researchers may need to directly contact interested participants, examine the age noted on participants’ social networking profiles or employ other age verification tools [59, 62, 70]. While researchers recognised that age misrepresentation is not confined to online research [59, 62, 71], one study argued that online users are inclined to falsify their age [70]. While the use of online consent procedures requires researchers to trust that participants are who they say they are [54, 64], it was acknowledged that postal consent procedures are subject to similar issues [59].

#### Participant vulnerability

A few articles noted that children and young people participating in online research should be “generally regarded as ‘vulnerable subjects’ requiring a heightened sensitivity to issues of gaining informed consent and ensuring confidentiality” [72, p. 554]; issues that were further amplified in the online sphere [63, 72]. Children are increasingly online and have greater knowledge, confidence and reliance upon digital technologies than adults [63]. However, they may not be aware of public and private boundaries and “are reluctant to remain anonymous” online [58, p. 65]. Children openly express themselves on the internet, revealing real names, locations and personal details, and may have different understandings of privacy and the permanence of online content [58, 72]. This is ethically challenging as children, more than adults, may freely share sensitive and personal information online without realising that others, including researchers, can access their posts and information [72].

Henderson et al. [72] provided further comment on the vulnerability of children in social media-based research and the array of complex ethical issues regarding obtaining informed consent and assuring privacy and confidentiality that may be difficult for adults to grasp, let alone children. As young people are generally disempowered in research, the authors suggest that researchers consult with children across all stages of the study so that they have greater control over the research process. They also suggest, as recommended by Sharkey et al. [71], that consent of children and young people in social media-based research should be ongoing or phased rather than a one-off occurrence, involving consultation with children at all stages of the research (from design to data collection, analysis and reporting).

Across the articles, there was little discourse on whether online research elevates risk among vulnerable people compared to offline research. One article stressed the importance of considering “whether individuals who choose to engage in internet research may be more vulnerable to safety concerns than their offline counterparts”, as there is some evidence that frequent internet users have poorer mental health than others [59, p. 1120]. Sharkey et al. [71] asserted that the context and medium of research itself can increase vulnerability. In their case, the online setting, the sensitive topic, the young age of participants, the lack of face-to-face interaction and the need to protect anonymity together created the ‘perfect storm’ of ethical issues, requiring careful consideration of ethical practice and how to maintain anonymity of a high-risk and vulnerable population.

#### Participant privacy, confidentiality and anonymity

Participant privacy, confidentiality and anonymity were the most commonly reported ethical concerns. These concerns are applicable to internet research across all disciplines, not just those involving families and children. Articles commented on the complexity of separating public and private domains online, particularly for social networking sites and online communities that encourage users to share information [45, 46]. The public nature of these sites was seen to be “inherently at odds with the confidential or anonymous nature of research” [63, p. 62]. While the terms and conditions of social media platforms usually indicate the potential for public disclosure, it is unlikely that users intend or expect their online information to be used by researchers [47, 72]. Additionally, confidentiality may be breached by participants inadvertently revealing their association with a study by writing on a comment board, ‘liking’ a study page, or adding a link to a study site on their own profile [45, 54]. Researchers are therefore reliant on participants understanding and using privacy settings [63, 72] to prevent unintended sharing of personal information and research involvement [47].

Additional issues concerned the risk of revealing participant identities via website breaches or deductive disclosure, and protecting participant privacy in studies that traced participants. Notably, and of benefit to other researchers, many articles reflected on how they sought to maintain participant confidentiality, privacy and anonymity in research conducted online and offered advice for future research, as discussed below.

Several articles discussed the risk of deductive disclosure of an individual’s identity for research conducted within ‘open’ discussion boards, forums and web pages [47, 58, 62, 64, 71]. Using a search engine to trace phrases or quotes from a research report or publication may reveal participants, thus breaching anonymity, confidentiality and privacy. Such risks may be reduced by omitting, anonymising or paraphrasing quotes [71], albeit potentially at the expense of the integrity of the original post. Researchers expressed concern that data that have been de-identified for reporting may not remain so with future technological developments [72].

Regarding online tracing, several articles describing the use of social media to trace and re-connect with lost participants questioned whether searching for a participant on social networking sites was a violation of online privacy [44, 47, 72]. One study that traced participants online stated that they only found information about participants from sites that individuals made publicly available [44]. The authors reasoned that this was essentially no different to finding individuals who list their name in public telephone directories or electoral rolls, since social media users are able to conceal or publicise their profiles. However, they noted that “there are legitimate concerns as to whether some (perhaps more vulnerable) individuals understand the full implications of making their personal details publicly visible” [47, p. 35], potentially to a much larger audience than intended [44, 47].

Three articles reported on participants’ experiences of being traced using social media. Two studies found participants were predominantly positive about the experience, although somewhat surprised about being re-contacted via social networking sites [44, 56]. Participants perceived Facebook to be a “more secure and private way of being contacted than by telephone or letter” [44, p. 32] and many were friendly and informal when contacted via Facebook. Conversely, one study found participants were cautious about engaging with researchers via social media as they were an older generation who lacked experience and confidence with that medium [47].

In general, using social media to trace participants was considered a valuable and worthwhile approach. However, authors were mindful that online tracing needs to be undertaken with care to ensure participant privacy and confidentiality. The majority of articles that used social media to trace participants (*n* = 4/5) discussed the ethical challenges they encountered when using this method [44–47]. They also provided advice on how to minimise breaches in participant confidentiality and improve study legitimacy, including how to contact participants privately, prevent individuals or researchers inadvertently revealing their involvement in a study and to ensure that a traced participant is a correct match.

Of all reviewed articles, a number emphasised the responsibility of the researcher in protecting online data and participant anonymity, confidentiality and privacy, and how this should be a top priority [43, 59, 71, 73, 74]. Authors stressed that researchers must be aware of participants’ expectations about privacy and confidentiality online [74] and be mindful of participants’ awareness and knowledge (or lack of knowledge) of internet technologies and privacy settings [47]. One article suggested that researchers have an “ethical duty to call attention to this lack of privacy” and to inform participants if their privacy and anonymity cannot be guaranteed online [43, p. 142]. Baker [43] used Facebook as a communication tool and source of data in ethnographic research with adolescents and required participants to add the researcher as a contact or ‘friend’. This presented an ethical challenge as all participants would be able to see one another on Facebook. To call attention to this, the consent form used Facebook terminology, explicitly stated that the anonymity and privacy of the participant could not be protected and instructed participants on how they could adjust their Facebook privacy settings to avoid inadvertent sharing of information.

Articles also highlighted that websites and platforms are rapidly changing as new features are developed [45, 54]. This requires researchers to be familiar with privacy settings and to keep up to date with changes that could affect their participants [44]. For example, advertising on social networking sites as a recruitment strategy requires researchers to know and understand how advertising features operate and what personal information is collected from participants interested in participating. While Facebook does not reveal individual user information to the advertiser, researchers may want to consider minimising the amount of information exchanged within such sites and direct interested participants to a secure external website where they have greater control over privacy [20, 54, 73].

#### Informed consent

A small number of articles discussed issues regarding informed consent when engaging participants online. Compared to some offline recruitment methods (such as recruiting participants face-to-face or by telephone), researchers have a “lack of immediate and real-time engagement with participants at the time of program enrolment online” [54, p. 1084] which may limit their ability to assess participant comprehension and ensure participants are truly informed [54, 64]. These concerns brought into question whether informed consent and implied consent can be trusted in online surveys. While one option is to ask participants to click a button at the beginning of an online survey to acknowledge their agreement and consent before being allowed to proceed, this method relies on participants having a sound understanding of the study and being competent to give informed consent [54, 64]. Obtaining verbal consent may be one way to help ensure participants are exposed to consent information prior to enrolment in online research [54, 59]. Telephone conversations allow researchers to use back-questioning techniques to help ensure participants have read and understood consent documents and the procedures, risks and benefits of a study.

As the ability to obtain face-to-face and verbal consent in internet research can be challenging, new and multiple strategies to ensure consent is informed are required. This may be particularly relevant if the research is anonymous or involves children and adolescents. One study reported how, during piloting of an online intervention, youth “didn’t express any concerns about why we were asking for sensitive information or what we would be doing with participant data” [54, p. 1086]. The authors concluded that this indicated children and young people are generally not interested in reading formal informed consent materials or may not have the ability to understand these concepts. Alternative, tailored information available in a number of different formats (e.g., audio-clips, images, video or bullet-point summary) and readily accessible throughout the duration of the study (e.g., sent via email, attached to the survey) could be considered [54, 63, 71]. Researchers could also give participants the opportunity to discuss the study online via a study page [54, 71]. However, one article did comment that providing extra measures and sources of information may not guarantee understanding, regardless of whether the research is conducted online or face-to-face [71]. Alternatively, researchers could employ a phased consent approach as reported by Sharkey et al. [71], where separate consent was taken from participants for study registration, participation and use of direct quotes, in order to increase participants’ understanding of the research and study protocol. The importance of maintaining consent when engaging research participants via social media was also discussed by Henderson et al. [72]. The authors noted how the dynamic nature of social media, including changes in the people involved in social networks over time, may alter the context of the initial consent. This raises questions regarding the ethical decision-making of researchers in social media-based research, and “whether there should be measures to check that consent continues to be given” [72, p. 551].

#### Disparities in internet access and sample representativeness

Some articles expressed concern over the ‘digital divide’, whereby internet-based recruitment strategies may limit the ability of researchers to reach groups disadvantaged by socioeconomic status or education, who lack internet access or digital literacy [36, 54, 59, 74]. This may introduce bias and limit the representativeness of the study sample [60, 75]. Conversely, some researchers argued that the digital divide was not a concern when recruiting young people online, given the high rates at which youth, including disadvantaged youth, use and access the internet [52, 54, 76].

Many articles commented on the representativeness of their online sample, with mixed findings. In some cases, samples recruited online were representative of the target population in terms of socioeconomic status and regional distribution [20, 52, 66] yet not regarding age or gender [20, 66, 77]. Online recruitment methods successfully engaged traditionally hard-to-reach populations such as regional, isolated or stigmatised groups [20, 55, 74] and allowed a broader sample to be recruited compared to offline methods of recruitment [55, 78]. Others found online recruitment was associated with under-representation of some populations (e.g., minority groups, low socioeconomic status populations) and over-representation of others (e.g., tertiary educated) [20, 49, 66, 79], although it was argued that similar sample biases are reported in offline recruitment methods [20, 66, 78]. One study reported similar demographic data for participants traced via Facebook and offline methods [46].

In most articles, sample representativeness was not addressed as an ethical concern but rather as a methodological limitation of the study design (e.g., [75, 80]). However, two articles did highlight the need to justify using the internet to engage research participants [54, 59]. They proposed that researchers should consider whether online methods are appropriate to engage and interact with research participants and which method (online or offline) will reach more of the target population. Care should also be taken to ensure that the chosen method of engagement is best for the participant, not the researcher, and to minimise the impact of disparities in internet access on participant inclusion or selection bias.

#### Incentives

Three articles discussed the ethical concerns associated with offering incentives in research [44, 62, 81], yet only one focused on incentives in online research. Boydell et al. [62] aimed to recruit youth to online focus groups and was interested in offering an incentive. The authors were however concerned that requesting personal details from potential participants could compromise anonymity. As available literature provided no guidance on this issue, they opted not to offer an incentive, which they state, might have contributed to their poor online recruitment rates.

#### Risks to researchers

Similar to participants, researchers may also be subject to privacy and safety risks when engaging participants online. While several articles reported interacting with participants online, for example via Facebook messaging features or discussion boards, only one study described the potential impact of this on the researcher and how they protected their personal privacy by creating a new Facebook account used solely for contacting participants [43]. Another study briefly acknowledged that sensitive research on adolescent self-harm may include risks for both participants and researchers, yet did not discuss this further [71].

### What ethical guidelines are available and being used to inform internet research?

#### Guidance offered by academic articles

The academic literature search identified a small number of articles offering ethical guidance when engaging research participants online. This is best illustrated in three case studies [54, 59, 71] where the authors list and present solutions to ethical issues encountered when recruiting young people on social networking sites and discussion forums. Other articles also provided ethical guidance by discussing the challenges encountered, the amendments made to study design and the strategies employed when using email in family research [74], recruiting participants into online focus groups [60, 62] and tracing research participants online [44, 47]. Several articles called for researchers to publish the methodological and ethical challenges they encounter when engaging research participants online and how these issues can be resolved [44, 59, 71, 72, 74], particularly when tracing participants [45, 47] and engaging adolescents [73].

#### Guidelines and resources cited by academic articles

Of the 58 research articles identified in the review, only three [35, 37, 82] described following internet-specific ethical guidelines when designing or undertaking their online research: these included professional guidelines produced by the Association of Internet Researchers (AoIR) [83] and the American Psychological Association (APA) [84], and practical guidance offered in academic literature [71, 85]. Some articles [20, 42, 54, 65, 86] reported consulting ethical guidelines produced by their institutional review board [56], country of origin (e.g., National Statement on Ethical Conduct in Human Research [27], Belmont Report [87]) or the World Medical Association [88]. However, none of these provide specific ethical guidelines on the conduct of internet research. While the Belmont Report clearly precedes internet research, other more recent guidelines do not refer to the internet or online research. A small number of internet-specific ethical guidelines were recommended by articles [59, 64, 71], including professional guidelines published by the AoIR [83, 89], APA [84] and the British Psychological Society (BPS) [90, 91]. Other internet-specific ethics resources were also briefly mentioned [92–96].

#### Available guidelines

A grey literature search identified a number of guidelines published from January 2006 to February 2016 specific to the ethical conduct of internet research. These included five professional guidelines developed by government bodies, societies and collaborative academic working groups (see Table 3) and ten university guidelines (see Table 4). Other resources included book chapters and websites.

**Table 3:** Professional guidelines for the ethical conduct of internet research.

Author / Affiliation	Title	Format	Ethical concerns discussed	Family/child-specific ethical concerns discussed	Specifically refers to recruitment, retention or tracing	Intended audience	Notes
Association of Internet Researchers (AoIR) (2012) [83]. This is an updated version of the 2002 guideline [89]	Ethical decision-making and internet research—Recommendations from the AoIR ethics working committee (Version 2.0)	Briefly describes ongoing dilemmas in internet-based research. Lists a broad yet comprehensive set of over 80 questions (grouped into 11 categories) to be considered	Informed consent, privacy, data security, harm, research with minors, among others	Identifies issues specific to minors and vulnerable persons, including exclusion, verifying identity and age, responding to harm and parental consent	Not directly	Primarily researchers, but applicable to review boards, ethicists and students	Provides a basic overview of ethical considerations applicable to all types of online research but does not provide practical solutions or recommendations
British Psychological Society (2013) [90]. This is an updated version of the 2007 guideline [91]. A revised version was published in 2017 [97]	Ethics guidelines for internet-mediated research	Offers guidance on interpreting the four main ethical principles in the context of online research, and what special considerations apply	Public-private domain, confidentiality and security of data, consent procedures, withdrawal and debriefing procedures, levels of researcher control, implications for scientific value and potential harm	Only states that offline parental consent procedures may be required in online research with underage or vulnerable participants	Yes—recruitment only	Researchers and ethics committees	Explores the ethical issues in more detail than AoIR guideline, particularly for recruiting participants online. Includes examples and recommendations to overcome issues
Secretary’s Advisory Committee on Human Research Protections (SACHRP) (2013) [98]	Considerations and recommendations concerning internet research and human subjects research regulations, with revisions	Discusses 20 regulatory considerations pertaining to internet research	Private versus public, obtaining informed consent, participant verification, sensitive and identifiable information, and harm minimisation	Discusses how research with minors raises concerns regarding age verification and parental consent procedures	Yes—recruitment only	Researchers and ethics committees	Primarily focuses on private versus public information online and legal jurisdiction. Provides recommendations and examples of how investigators should approach online research including online recruitment
The National Committee for Research Ethics in the Social Sciences and the Humanities (NESH) (2014) [99]. This replaces the former version from 2003 [100]	Ethical guidelines for internet research	Reviews key ethical issues common to internet research and how these relate to fundamental ethical principles	Private versus public, informed consent, privacy and confidentiality, children’s right to protection, use of quotes and the regard for third parties	Discusses how online research with children raises special issues as children actively use the internet, often beyond adult control. States that parental consent and age verification are important but difficult online. In some cases, it may be better to use offline research tools	Not directly	Researchers	Does not give explicit guidance on how to overcome issues, but highlights what researchers need to consider for both passive and active participation
Clark et al; Carlton Connect Initiative (2015) [101]	Guidelines for the ethical use of digital data in human research	Provides an extensive discussion of ethical issues for researchers and provides practical approaches for ethics committees. Provides an extensive list of resources regarding internet research	Consent, privacy and confidentiality, ownership and authorship, governance and data sharing	Only states that vulnerable, underage people may be unknowingly included in online research	Not directly	Researchers and ethics committees	Predominantly focuses on the use of ‘big data’ in research

Note: The guideline published by the American Psychological Association [84] cited by articles in the review is not included in this table as it was published before 2006 and was considered outdated.

**Table 4:** University guidelines for the ethical conduct of internet research.

Author / Affiliation	Title	Format	Ethical concerns discussed	Family/child-specific ethical concerns discussed	Specifically refers to recruitment, retention or tracing	Notes
Brunel University [102]	Guidelines for research on the internet	Briefly discusses context, principles and issues of online research	Outlines 3 issues (human participants; private/public; data/persons)	No	No	Reiterates AoIR guidelines [83], attached as appendix
Florida Atlantic University [103]	Guidelines for computer & internet-based human subjects research	Discusses online instruments and their integrity, identifies several ethical considerations particularly regarding online data. Offers procedural guidelines. Includes sample consent form	Discusses informed consent, data collection and data storage/disposal	Recommends limiting online research with minors to minimal risk research that qualifies for waiver of parental consent due to age verification concerns	Yes—recruitment	Similar to other university guidelines [104–106]
Pennsylvania State University [104]	IRB guidelines for computer- and internet-based research involving human participants	Procedural guideline and recommendations	Discusses informed consent, server administration, data collection and data storage/disposal	No	Yes—recruitment	Similar to other university guidelines [103, 105, 106]
Queensland University of Technology [107]	Internet and social media	Provides detailed discussion of ethical considerations and concerns. Offers procedural guidance and recommendations for online recruitment, survey tools and consent	Discusses potential biases, risks, vulnerable participant groups, public versus private space, consent, data collection, data mining	Discusses children’s vulnerability and naivety online, emphasises responsibility of research to consider issues of consent, privacy and risk	Yes—recruitment	
The Royal Children’s Hospital Melbourne [108]	Social media: use in research	Procedural guidelines when preparing research ethics application	Briefly outlines methodological considerations of recruiting, communicating and tracing participants via social media	No	Yes—recruitment, retention and tracing	
University of Bedfordshire [96]	Ethical guidelines for the online researcher	Provides a guide of online etiquette, particularly when collecting data online from forums/boards. Includes FAQ section	Discusses electronic consent, pseudonyms, researcher safety	No	Yes—recruitment	Provides extensive list of resources and guidance documents
University of California, Berkeley [109]	Internet-based research	Provides detailed discussion of ethical considerations and concerns, as well as procedural guidance	Offers specific guidance and information on recruitment, informed consent, data collection and data security	States that online research with minors should obtain child assent and parental consent, unless a waiver is appropriate. Refers to the Children’s Online Privacy and Protection Act (COPPA).	Yes—recruitment	Provides reference list for further information
University of Connecticut [105]	Guidance for data security and internet-based research involving human participants	Procedural guideline and recommendations	Discusses data collection and security, data collection software, data storage/disposal and informed consent	No	Yes—recruitment	Similar to other university guidelines [103, 104, 106]
University of Rochester [106]	Guideline for computer and internet based research	Detailed procedural guideline and recommendations. Discusses ethical concerns and strategies	Discusses recruitment; privacy, anonymity and confidentiality; data validity and participant misrepresentation; informed consent process; data collection, server administration, and data storage/disposal	Briefly discusses age verification and child assent/parental consent requirements. Refers to COPPA	Yes—recruitment	Similar to other university guidelines [103–105]
Webster University [110]	Guidelines for internet research	Provides definitions of online research methods, discusses considerations and concerns, and provides procedural guideline and recommendations	Discusses consent processes; confidentiality and identifying information; data storage; and minimal risk research	No	Yes—recruitment	

Regarding professional guidelines, the most widely cited ethical guidelines for internet research are those published by the AoIR [83] and the BPS [90, 97]. These offer broad advice to help researchers and research ethics committees make ethical decisions across all forms of internet research. They do not prescribe a code of practice or a ‘rule book’ but instead advocate for a flexible, case-based approach. Both recognise that the guidelines should not be considered as complete and that ethical decision-making will continue to evolve as new online tools are developed and new issues emerge. Produced in the USA, the AoIR guidelines briefly describe ongoing dilemmas in internet-based research before listing a set of guiding questions that should be considered when undertaking or reviewing online research methods. The document provides a basic overview of ethical considerations applicable to all types of internet research. While it does not specifically mention recruiting, retaining or tracing participants, it does raise key ethical concerns applicable to recruitment and outlines particular issues that may arise in online research with minors or vulnerable persons. Many of the ethical considerations listed in the AoIR guidelines are explored in more detail in the BPS guidelines, particularly regarding active recruitment of research participants. The BPS guidelines also provide recommendations and practical advice to guide ethical decision-making. These guidelines do not refer to child-specific ethical issues other than briefly stating that parental consent procedures will likely need to be obtained offline.

The grey literature search identified other professional guidelines not cited by articles identified in the review. Produced in Australia, Clark et al. [101] provide comprehensive advice for the use of digital data in research. Predominantly focusing on research using ‘big data’ rather than the direct involvement of participants, each issue is discussed in detail with examples and questions for consideration. The document also offers guidance to research ethics committees about ethical review processes. Produced in Norway, The National Committee for Research Ethics in the Social Sciences and the Humanities (NESH) guidelines [99] provide a brief discussion of ethical issues common to internet research. This does not give explicit guidance on how to overcome these issues but highlights how researchers must keep these issues in mind while continuing to follow general, fundamental ethical principles. The guidelines also include a section on protecting children in online research and emphasise the importance of obtaining parental consent and verifying age. Similar issues are raised in guidelines produced by the USA Secretary’s Advisory Committee on Human Research Protections (SACHRP) [98], where regulatory and ethical considerations pertaining to internet research are discussed, including online recruitment.

The grey literature search identified several universities, predominantly within the USA and UK, providing publicly accessible guidance on the ethical use of the internet in research. The content of the university guidelines varied widely and included comprehensive guidelines (e.g., [96, 109]), general procedures and policies regarding recruitment, data collection and data storage (e.g., [104]) and resources that reiterate the AoIR guidelines (e.g., [102]). Guidelines developed by universities were often aimed at researchers and developed by ethics committees and review boards, to provide step-by-step procedural guidance to assist with ethical review processes. The search also identified one guideline produced by a university hospital [108] providing ethics-based recommendations and protocols for social media-based research, including online tracing. While some guidelines discussed ethical issues specific to online research with children, most did not. Two guidelines from universities in the USA reiterated the Children’s Online Privacy and Protection Act (COPPA), a federal law that requires verifiable parental consent if researchers collect personal information from children under 13 years of age.

Other resources describing the ethical issues of online research were also identified in the search. Several book chapters were identified [92, 94, 95, 111]. These provide examples of online research methods and discuss common ethical issues, rather than offering procedural guidance. An online entry in the Stanford Encyclopaedia of Philosophy [112] provides a detailed discussion of key ethical issues in internet research including a verifying age and obtaining consent with minors. Several research ethics blogs and blog posts [113–115] and an online training program [93] were also found.

## Discussion

This review is the first attempt to identify and integrate literature on the ethics of using the internet to engage participants in family and child research. The review is timely in the context of changing communication patterns, increased social mobility and a research environment with limited funding. Communicating via the internet has rapidly emerged as the ‘new normal’ in everyday life; a development that has extended to the research world. Researchers (and, by extension, ethics committees) have a number of ethical concerns when engaging participants online. Some of these apply when engaging *any* research participant online, such as protecting participant privacy and confidentiality, obtaining consent and disparities in internet access. Engaging minors online raises additional ethical concerns and considerations, including parental consent, age verification and participant vulnerability. While these issues highlight some overlap between offline and online methods of participant engagement, the ‘visible’, far-reaching and dynamic nature of online platforms, especially social media, can inflate risk and introduce new challenges. Clearly, there are specific nuances of the online environment that the research community should be aware of. However, as highlighted in this review, many research articles do not report the ethical issues associated with engaging research participants online, and few refer to internet-specific ethical guidelines.

The review identified three unique ethical issues to be considered when engaging minors in research online. The first relates to the ethical and practical complexities of obtaining parental consent online. Parental consent serves to respect children’s developing autonomy and to provide for their protection [27], although in some cases may not be appropriate or protective. Inconsistent approaches and ambiguities in defining an adolescent, a mature minor and minimal risk research have led to extensive debate on the ethics and value of parental consent [116–118]. As recognised in this review, parental consent regulations differ by location, participant age, and research design and context, and are subject to case-by-case interpretation by ethics committees [119, 120]. Academic researchers used a variety of methods to record parental consent. Limitations were reported for both offline and online procedures, and many authors noted a lack of guidance on ethical best practice. This was reflected in the grey literature search, which provided no formal guidance on how to document parental consent online. Instead, professional guidelines suggest researchers utilise offline consent procedures [90, 99] while one university guideline recommends limiting online research with minors to minimal risk research that qualifies for a waiver of parental consent [103]. Guidelines on ethical best practice for obtaining parental consent online are clearly needed. In the interim, researchers and ethics committees may seek guidance from literature identified in this review [59] and elsewhere [4, 121].

The second unique issue of verifying participant age was an important yet challenging aspect of engaging minors in research online as young people may misrepresent their age to participate. Articles suggested several strategies to verify age, including directly contacting the participant or their parent. However, depending on the nature and context of the study, this may be inappropriate or unfeasible. Others suggested validating participant age by reviewing social media profiles. This raises further concern for participant privacy and may not be a valid approach if the minor has falsified their age (e.g., Facebook users must be at least 13 years old to create an account). Articles did not explicitly discuss the ethical implications of inadvertently engaging younger or underage participants in research, despite the potential for them to be exposed to sensitive or age-inappropriate content. Guidance on this issue is mixed: one guideline [98] suggests researchers include fact-checking measures in online research instruments or employ age verification software; others suggest limiting online research with minors to minimal risk research [103] or to conduct the research offline [99].

The third issue identified in the review relates to children’s vulnerability in online research. Young people are often referred to as ‘digital natives’: a younger generation born into a ubiquitous digital environment who have used the internet, social media and mobile devices from an early age [122]. However, young people may also be digitally naïve [123], openly disclosing personal and potentially sensitive information without apparent concern for their privacy, without understanding or considering the permanence or far-reaching nature of online content, and without intending for their information to be used by others. Internet users are largely unaware of targeted advertising practices and the extent to which their online information and behaviours are collected and used by third parties [124], including researchers [125]. Privacy and data use policies are outlined in click-to-agree contracts and in website Terms of Service. Few users read or comprehend these often lengthy and indecipherable agreements and few understand how to protect their privacy online [126]. Discourse on how these issues apply in research is growing [127], yet literature exploring whether privacy risks are exacerbated for minors and vulnerable groups online is limited. Due to a lack of maturity, understanding, interest and/or digital literacy, it is possible that children and adolescents are less inclined than adults to read privacy and data use policies and understand how their online information is collected and used. Yet, this demographic group are prolific internet and social media users, and are increasingly encouraged to share their personal information [128]. This brings into question the appropriateness of researchers using this information to target individuals to participate in research, as is increasingly occurring.

Concerns were also raised about the possibility of participants, potentially those who are young or vulnerable, unwittingly ‘outing’ themselves as research participants online. This may occur due to a lack of knowledge regarding privacy settings, features and the reach of online platforms. While this may be no different to offline research where participants may disclose their participation to others, the audience is much more far-reaching in the online space and individuals may lose control of their information once they ‘release’ it online. These concerns require researchers to be transparent and openly inform prospective participants about potential risks to their privacy, confidentiality and anonymity online, as suggested by articles included in the review [43] and in recent literature [125, 129].

An additional issue that received minimal attention in the review but which warrants further discourse is whether participants, particularly minors, are sufficiently informed in online research. One article noted that adolescents were generally uninterested in reading study information and informed consent materials [54]; an observation that has been reported by others engaging participants online and offline [130, 131]. Children and adolescents may also misunderstand the information that is presented to them, which can be difficult for researchers to detect [132]. While researchers are advised to spend time with prospective participants and orally explain the research to them [133, 134], this is often not possible or practical in online research. That young people are uninterested in consent materials or cannot understand them is ethically problematic in any research setting. These concerns may escalate in online research as parental consent is commonly waived, participants are separated physically and temporally from the researcher, and the immediacy of online platforms may prevent careful consideration of participation.

Despite recruiting participants online, many articles in this review obtained consent via traditional means (e.g., by post, face-to-face). The ongoing reliance on offline consent procedures may reflect concern for participant comprehension in online settings. However, the inability to assess whether participants are truly informed is not confined to online research (e.g., postal surveys), and articles often compared the deficiencies of online consent procedures against an idealised view of the communication than can, but often does not occur in face-to-face consent. Articles suggested several strategies to improve participant comprehension online, including video conferencing and phased consent. Some recognised the advantages of obtaining verbal consent and assessing comprehension via telephone, although we question the viability of this approach with large samples and its utility in anonymous research. Emerging evidence indicates that testing adolescents on their research rights and risks during the online consent process can improve study comprehension [135]. Further discourse on the effectiveness of alternative online approaches are warranted.

Our review has identified several insights and gaps in the academic literature that are worth noting. Firstly, while researchers have sought new ways to engage participants online, a continued reliance on traditional offline recruitment methods remains. The use of offline and online methods may help overcome technological barriers such as the digital divide but may also reflect uncertainty regarding the effectiveness of solely using the internet. Clear evidence for effectiveness is currently hampered by an absence of, or lack of detail in, reporting the success of recruitment, retention and tracing methods. Reporting ethics approval processes and how consent is obtained online could also be improved. The absence of this information does not ease concerns regarding the validity and credibility of online recruitment and online consent. We encourage researchers to report response metrics and/or recruitment source data as well as consent procedures. Researchers reporting results for internet surveys may wish to refer to Eysenbach’s checklist [136].

Over half of the articles included in the review did not discuss ethical issues beyond general ethical procedures. The limited discussion of ethical concerns aligns with that reported by Henderson et al. [72], who reviewed studies using social media to involve children or young people in research. They found articles were similarly silent on ethical issues. It is unclear why ethical issues are not acknowledged or at least not reported. It is possible that researchers view the ethical issues of engaging participants online as similar to offline methods. This has implications for research integrity and participant safety as some issues and risks posed online are different to traditional offline methods. Alternatively, researchers may not expect to comment on ethical issues if their study has received approval by a governing ethics committee, and publication word limits may preclude detailed description of ethical issues. Like Henderson et al. [72], we do not consider studies that are silent on ethical issues as unethical. Given the increasing use of online methods, the research community will greatly benefit from more detailed documentation of the ethical and methodological issues encountered or considered and the actions taken to address them.

We recognise that many of the ethical concerns identified in this review pertained to online recruitment. Using the internet to retain or trace participants was not as widely used or at least not commonly reported. This could be due to limitations in our search strategy, as articles may not explicitly refer to retention and tracing in their title, abstract or key words. Alternatively, it could reflect the smaller number of longitudinal studies (which require retention and tracing) relative to cross-sectional studies, or that use of the internet in this way is an emerging method not yet in widespread use. Nonetheless, it is surprising that so few retention and tracing studies were identified given the recognised difficulty in maintaining research cohorts in longitudinal research [137, 138]. It is unclear whether the internet is not used for this purpose because researchers and/or ethics committees view it as problematic and unethical, or because they are unfamiliar with such methods. Most articles that reported online retention used email to maintain contact with participants, which was not considered to be ethically concerning. Other online retention strategies involving social media have been reported, such as researchers ‘friending’ participants on Facebook [139, 140]. While these strategies can be effective at retaining adolescents in research, they raise ethical issues regarding participant confidentiality, the participant-researcher relationship, and potentially accessing information beyond what participants originally consent to. Most tracing studies identified in this review used social media to search for participants lost to follow-up, with many authors providing comment on the ethical and methodological considerations undertaken. Researchers interested in tracing participants via social media in the future will benefit from consulting these articles and other relevant literature [25, 141]. Given its effectiveness, tracing participants via social media is likely to become more popular among researchers, but the lack of ethical guidance for this method is concerning.

Lastly, we show that most researchers do not refer to internet-specific ethical guidelines when reporting online recruitment, retention or tracing methods. Previous research has identified a perception from researchers and ethics committee members that there are few guidelines available to inform internet research [142]. Our review shows internet-specific ethical guidelines are available, yet researchers are largely unaware of them. Existing guidelines provide an overview of common ethical concerns, many of which are identified in this review, that are applicable when recruiting participants online. They do however vary in their content, scope and guidance, which may require researchers and ethics committee members to familiarise themselves with each resource and use the guidelines collectively when planning or reviewing online research. Most guidelines do not specifically comment on actively engaging research participants online, particularly for retaining or tracing participants via social media or for recruiting minors online.

Since conducting our literature searches in early 2016, additional internet-specific guidelines have emerged [143–147]. These guidelines generally focus on social media content as data rather than directly engaging participants online. Guidelines specifically addressing social media recruitment have also been developed [125, 148], providing case studies and recommendations for both researchers and ethics committees. Given the dynamic and diverse nature of the internet, the majority of internet-specific ethical guidelines are purposefully broad, flexible and applicable to a range of online research methods. While there is a strong argument for guidelines to remain broad, a more structured procedural guideline that specifically and clearly outlines common methodological and ethical considerations when recruiting, retaining and tracing participants, including children, online will be well received.

### Limitations

This review has several limitations. While we searched six academic databases and aimed to be inclusive with terminology, there may be relevant studies and articles that were not captured by the search strategy. For example, articles may describe ethical issues without referring to ‘ethics’ in their title, abstract or key words. Studies using online retention and tracing strategies may also not explicitly report this, as discussed, which may account for the few retention and tracing studies identified. While Facebook is a common research tool, the high proportion of studies using Facebook compared to other social media platforms may be due to this being the only platform specifically named in our search strategy. We recognise articles in other disciplines not included in this review would inform family and child research. We also recognise the limitations of the grey literature search, which was neither exhaustive nor systematic, with results limited to the first 100 hits. Google is not well suited for complex search queries and results are retrieved by popularity; therefore, relevant ethical guidelines may have been missed.

### Conclusions

This scoping review provides a comprehensive overview of the ethical issues that arise when engaging participants in family and child research online and the available ethical guidance on online research. It serves as a useful resource for researchers and ethics committee members who are considering the ethical appropriateness of research in this context. All in all, using the internet and social media to recruit, retain and trace participants is possible, but must be harnessed appropriately. This requires researchers and ethics committees to be cognisant of a range of ethical issues, including the unique challenges associated with engaging minors online, and to carefully consider how these issues apply when planning, conducting and reviewing research.

Our review shows that researchers consider themselves primarily responsible for ensuring internet research is conducted ethically, yet it remains unclear to what extent researchers are aware of and appropriately informed of the specific ethical issues associated with engaging research participants online, particularly minors. Ethical guidelines are essential to consistent and high-quality decision-making, yet the lack of reported use of available guidelines and the scarcity of academic literature describing ethical concerns means research and ethical review are potentially occurring without suitable guidance. Increasing the research community’s understanding of the contextual and ethical challenges of engaging participants online is clearly required. This can be achieved by encouraging broad dissemination and use of current guidelines and resources (e.g., [83, 97, 125]) across research institutes and ethics committees. We also encourage researchers to provide more detail in academic articles when reporting on the internet as a research tool, including response metrics, ethical approval and consent procedures, and methodological and ethical considerations, particularly for research with children and adolescents. This review brings the ethical issues and guidelines presented in this review to the attention of the wider research community and promotes further discourse regarding the ethical conduct of recruiting, retaining and tracing participants online in family and child research.

## Supporting information

S1 File Additional search limits applied to Scopus database. (DOCX)

S2 File Academic articles data. (XLSX)
