# Supplementary material for: Ethical issues in using the internet to engage participants in family and child research: A scoping review
Source: PLoS One. 2018 Sep 27;13(9):e0204572. doi: 10.1371/journal.pone.0204572 (PMC6160098; doi:10.1371/journal.pone.0204572)
Supplement: S1 File — (DOCX) [file pone.0204572.s001.docx]

**S1 File. Additional search limits applied to Scopus database.**

(LIMIT-TO (SUBJAREA, "SOCI”) OR LIMIT-TO (SUBJAREA, "PSYC”) ) AND (LIMIT-TO ( SRCTYPE, "j”) ) AND (LIMIT-TO (EXACTKEYWORD, "Ethics”) OR LIMIT-TO (EXACTKEYWORD, "Methodology”) OR LIMIT-TO (EXACTKEYWORD, "Child”) OR LIMIT-TO (EXACTKEYWORD, "Internet”) OR LIMIT-TO (EXACTKEYWORD, "Psychology”) OR LIMIT-TO (EXACTKEYWORD, "Informed consent”) OR LIMIT-TO (EXACTKEYWORD, "Research”) ) AND (EXCLUDE (EXACTKEYWORD, "Middle Aged”) OR EXCLUDE (EXACTKEYWORD, "Middle aged”) OR EXCLUDE (EXACTKEYWORD, "Aged”) OR EXCLUDE (EXACTKEYWORD, "Human relation”) OR EXCLUDE (EXACTKEYWORD, "Organization and management”) ) AND (EXCLUDE (EXACTKEYWORD, "Legal aspect”) OR EXCLUDE (EXACTKEYWORD, "Health care policy”) OR EXCLUDE (EXACTKEYWORD, "Policy”) OR EXCLUDE (EXACTKEYWORD, "Curriculum”) OR EXCLUDE (EXACTKEYWORD, "Autism”) OR EXCLUDE (EXACTKEYWORD, "Treatment Outcome”) OR EXCLUDE (EXACTKEYWORD, "Data Collection”) OR EXCLUDE (EXACTKEYWORD, "Human immunodeficiency virus infection”) OR EXCLUDE (EXACTKEYWORD, "Patient Selection”) OR EXCLUDE (EXACTKEYWORD, "Genetics”) OR EXCLUDE (EXACTKEYWORD, "HIV Infections”) OR EXCLUDE (EXACTKEYWORD, "Physiology”) OR EXCLUDE (EXACTKEYWORD, "Doctor patient relation”) OR EXCLUDE (EXACTKEYWORD, "Medical student”) OR EXCLUDE (EXACTKEYWORD, "Politics”) OR EXCLUDE (EXACTKEYWORD, "Social justice”) OR EXCLUDE (EXACTKEYWORD, "Physician-Patient Relations”) )
